# EARLY gestational exposure to isoflurane causes persistent cell loss in the dentate gyrus of adult male rats

**DOI:** 10.1186/s12993-017-0132-5

**Published:** 2017-12-26

**Authors:** Arvind Palanisamy, Gregory Crosby, Deborah J. Culley

**Affiliations:** 1 Department of Anesthesiology, Perioperative and Pain Medicine, Brigham and Women’s Hospital, Harvard Medical School, Boston, MA USA; 2 Present Address: Department of Anesthesiology, Washington University School of Medicine, St. Louis, MO USA

**Keywords:** Anesthesia during pregnancy, Anesthetic neurotoxicity, Maternal anesthesia, Spatial working memory, Stereology, Hippocampus, Dentate gyrus, CA1 region

## Abstract

**Background:**

Our previous research showed that 4 h of maternal anesthesia with isoflurane during early gestation in pregnant rats leads to a deficit in spatial memory of adult male offspring. Because spatial memory is predominantly a hippocampally-mediated task, we asked the question if early gestational exposure to isoflurane affects development of the hippocampus in the offspring.

**Findings:**

Previously behaviorally characterized adult male rats that were exposed to isoflurane during second trimester were sacrificed at 4 months of age (N = 10 and 13, control and isoflurane groups, respectively) for quantitative histology of hippocampal subregions. Sections were stained with cresyl violet and the total number of cells in the granular layer of the dentate gyrus and the pyramidal cell layer in the CA1 region were determined by a blinded observer using unbiased stereological principles and the optical fractionator method. Data were analyzed using Student’s *t* test; P < 0.05 was accorded statistical significance. Stereological examination revealed 9% fewer cells in the granular layer of the dentate gyrus of isoflurane-exposed adult rats compared to controls (1,002,122 ± 84,870 vs. 1,091,829 ± 65,791, respectively; Mean ± S.D, *P = 0.01). In contrast, there were no changes in the cell number in the CA1 region, nor were there changes in the volumes of both regions.

**Conclusions:**

Our results show that maternal isoflurane anesthesia in rodents causes region-specific cell loss in the hippocampus of adult male offspring. These changes may, in part, account for the behavioral deficits reported in adult rats exposed to isoflurane in utero.

## Main text

### Introduction

Despite robust evidence for anesthesia-induced neurotoxicity in postnatal rodent models [1–4] not much is known about the effects of maternal anesthesia on the fetal brain. This is significant because most non-obstetric surgeries and fetal intervention procedures that are performed during the second trimester require maternal anesthesia [5]. Furthermore, the robust neuronal proliferation and migration observed during this period [6] is propelled by gamma-amino butyric acid (GABA) and glutamate [7–9] precisely the same neurotransmitter mechanisms by which anesthetic agents exert their effects [10]. Thus, clinical necessity and practice may inadvertently place the fetal brain at risk by disrupting the trophic milieu that propels early neurodevelopment [11]. We previously showed that a single 4 h exposure to isoflurane during mid-gestation impairs acquisition of spatial memory in the adult male offspring [12]. This suggested that prolonged and unphysiological stimulation of these neurotransmitter mechanisms can be detrimental to fetal brain development. Because acquisition of spatial memory is reliant on the structural integrity of the hippocampus [13], we hypothesized that early gestational exposure to isoflurane during maternal anesthesia will derail normal development of the hippocampal formation. Therefore, in the present study, we assessed the changes in neuronal number in the pyramidal cell layer of CA1 region and the granule cell layer of the dentate gyrus of the behaviorally dysfunctional adult male rats that were exposed to isoflurane in utero.

### Materials and methods

Experiments were conducted on timed-pregnant Sprague–Dawley rats (Charles River Laboratories, Inc, Wilmington, MA) on embryonic day 14 (E 14), a period that corresponds to the second trimester of human pregnancy [14], and their male offspring as described previously [12]. We chose 2–3 dams/treatment condition based on results from our previous study that indicated absence of maternal–fetal interaction during statistical analyses. Briefly, animals randomized to anesthesia received 1.4% isoflurane in 100% oxygen for 4 h whereas control animals received 100% oxygen for 4 h in identical anesthetizing chambers. The animals breathed spontaneously and the isoflurane concentration was measured continuously with an agent analyzer (Datex, Tewksbury, MA; Ohmeda, Madison, WI). During isoflurane anesthesia, maternal blood pressure was measured non-invasively every hour, rectal temperature was maintained at 37 ° ± 0.5 °C with heating pads, and venous blood gas and plasma glucose were measured at the end of the anesthetic. Following anesthesia, the anesthetized and control dams recovered in 100% oxygen for 20 min following return of righting reflex. Maternal weight was monitored daily until delivery of the pups on gestational day 22 [12]. All experiments had the approval of the Institutional Animal Care and Use Committee of Longwood Medical Area (Boston, MA, USA), and appropriate care was taken to minimize the number of animals used and their suffering.

#### Preparation for stereology

**Fig. 1:**
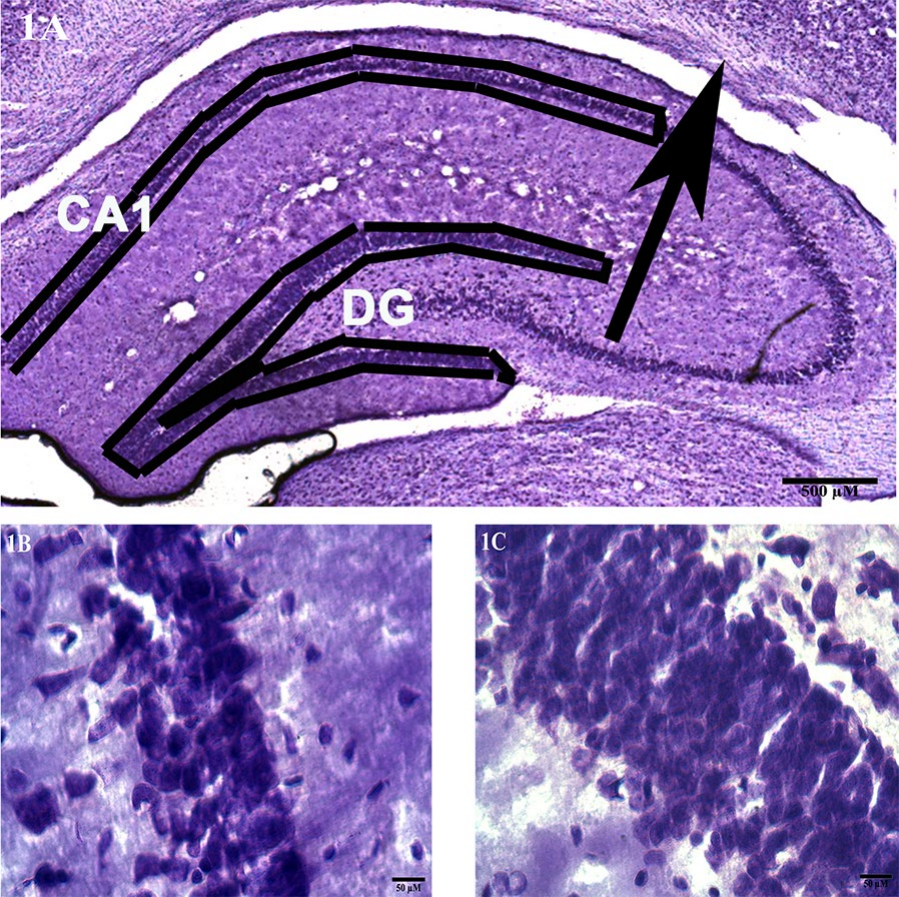
Sample microphotographs to illustrate the regions investigated. **A** A 2× image of a Nissl-stained section from a control rat demonstrating the CA1 area and the dentate gyrus in the right hippocampus. The arrow indicates the point of intersection to determine the CA1 area. **B** A 63x image of a Nissl-stained section from a control rat showing pyramidal cell bodies in the stratum pyramidale of the CA1 area, and **C** A 63x image of a Nissl-stained section from a control rat showing a densely packed granule cell layer of the dentate gyrus. Scale bars as indicated

Following behavioral characterization, male rat offspring (N = 10 and 13 in control and anesthesia groups, respectively) were anesthetized and perfused intracardially using 0.9% saline followed by a fixative containing 4% paraformaldehyde in 0.1 M phosphate buffer (pH 7.4) on postnatal day 120. The brains were removed and post-fixed in the same fixative at 4 °C overnight and cryoprotected in 30% sucrose at 4 °C overnight until sectioning. Coronal sections were cut at 50-μm thicknesses using a Leica 1800 CM cryostat at – 20 °C (Leica Microsystems Inc., IL, USA). Every section that contained the hippocampus was collected in a rostral-to-caudal serial order in phosphate buffer (0.1 M, pH 7.2) and the regions of interest, i.e., CA1 and the dentate gyrus (Fig. 1A–C), were delineated using a stereotaxic rat brain atlas. Approximately 72 sections/brain that included the regions of interest were collected, stained with 0.1% aqueous cresyl violet (Sigma, St. Louis, MO), dehydrated in ethanol, cleared in xylene and cover-slipped with Permount mounting medium (Fisher Scientific, PA, USA).

#### Stereological quantitation

Every 12th section of the series was chosen for stereological analysis because we established that every 12th section, when compared to every 8th or 10th section, produced reliable estimates of cell numbers in the dentate gyrus as well as the CA1 area. Moreover, this approach has been validated by an elegant study done previously to study anesthetic neurotoxicity in postnatal rodents [15]. The first section of the series was chosen at random, based on the roll of a die. The stereology equipment consisted of a light microscope (Nikon E2000) connected to a CCD camera (Microfire; Optronics, CA, USA), motorized X–Y stage (Ludl Electronics Products, NY, USA), z-axis indicator (MT12 microcator; Heidenhain, Traunreut, Germany) and a computer running Stereo Investigator software (Micro- Brightfield, Inc., VT, USA). Briefly, outlines of the dentate gyrus and the CA1 were drawn at 2× magnification in the right hippocampus from each section. Systematic random sampling was performed by randomly translating a 110 × 250 μm^2^ grid onto the section of interest. A 35 × 35 × 10 μm^3^ optical dissector was created at each sampling site using a 63× objective (oil; numerical aperture, 1.3). The average mounted thickness was 15-μm with a guard zone of 2-μm. Neurons were identified by a clear nuclear profile containing a nucleolus and Nissl substance in the cell body, as the focus went through the section from one grid to the other. Cell counts were limited to the pyramidal cell layer in the CA1 region and the granule cell layer (GCL) of the dentate gyrus. The software package converted the counts into total numbers of cells, which were subsequently multiplied by 2 to obtain estimates of cell numbers for both hemispheres. The total volumes of the GCL of the dentate gyrus and the CA1 pyramidal cell layer in the right hippocampus were estimated in the same sections used to determine the total neuronal count using the Cavalieri principle and the Stereo Investigator software. Briefly, the layers of interest were outlined in each section at 4× magnification and an unbiased point counting method was employed. The area of the grid associated with each point on the section, A/P, was 50 µm^2^, and a value of 7 was assigned as the shape factor. The volume was calculated using the formula V = ∑P X A/P X T, where ∑P is the sum of the cross-sectional area of the points inside the outlined borders, T is the cut thickness of the Section (50-μm), and A/P the area associated with each point (50 µm^2^). The Gunderson coefficient of error (CE), a measure of the quality of the estimates, was < 10% in the stereology runs for both cell numbers and volumes. Data were analyzed using Student’s t-test and expressed as mean ± S.D; P < 0.05 was accorded statistical significance.

### Results

**Fig. 2:**
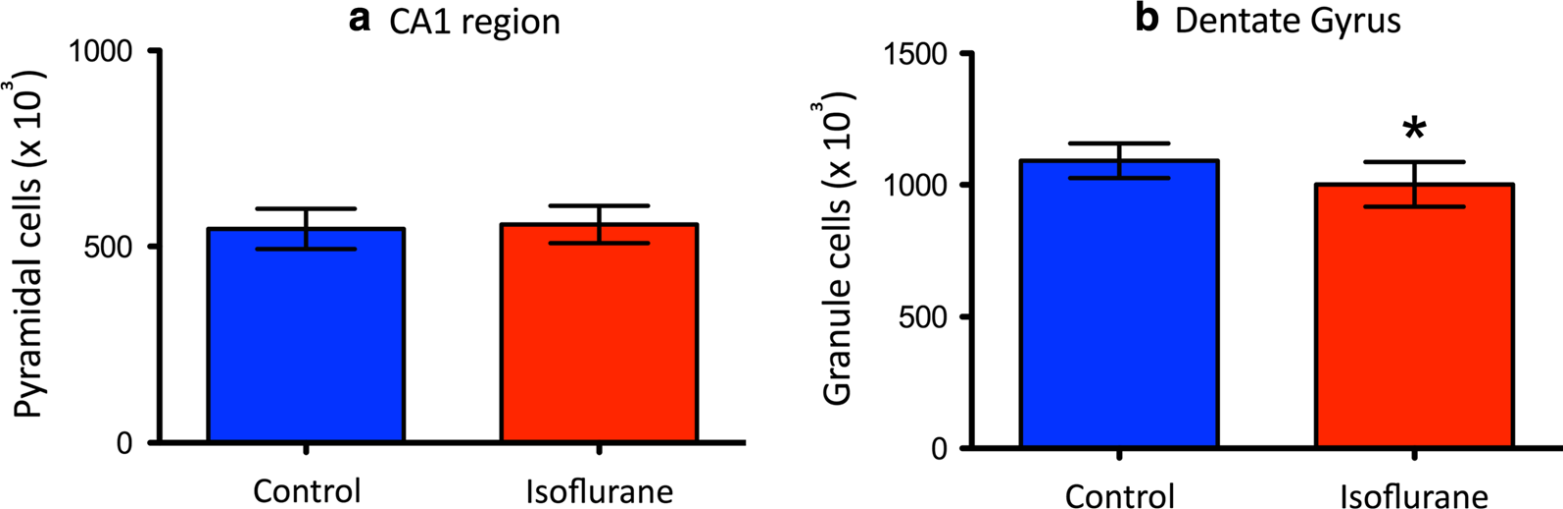
Gestational exposure to isoflurane resulted in region-specific cell loss in the adult hippocampus. No changes were detected in the pyramidal layer of the CA1 area of adult male rats following isoflurane exposure in utero (N = 13) compared to control rats (N = 10) (**a**). However, isoflurane exposure at E14 (N = 13) was associated with a significant decrease in the number of cells in the granule cell layer of the dentate gyrus (*P = 0.01) compared to control rats (N = 10) (**b**). Data were analyzed using Student’s t-test and expressed as mean ± S.D

Isoflurane anesthesia was well tolerated by the dams with no evidence for physiologic perturbance. There were no differences in the male litter size (6 ± 2.8 vs. 5.3 ± 1.2; P = 0.72) nor were there any differences in the weights of the adult male offspring (370 ± 29 vs. 370 ± 19 g; P = 0.92) between control and isoflurane groups, respectively. Stereological quantification revealed no differences in pyramidal cell number of the CA1 region between control and gestationally isoflurane-exposed groups (545,375 ± 51,272 vs. 556,493 ± 47,726, respectively; P = 0.60) (Fig. 2a). However, adult male rats exposed to isoflurane during early gestation showed a significant decrease in the number of cells in the granule cell layer of the dentate gyrus compared to control rats (1,002,122 ± 84,870 vs. 1,091,829 ± 65,791, *P = 0.01) (Fig. 2b).

**Fig. 3:**
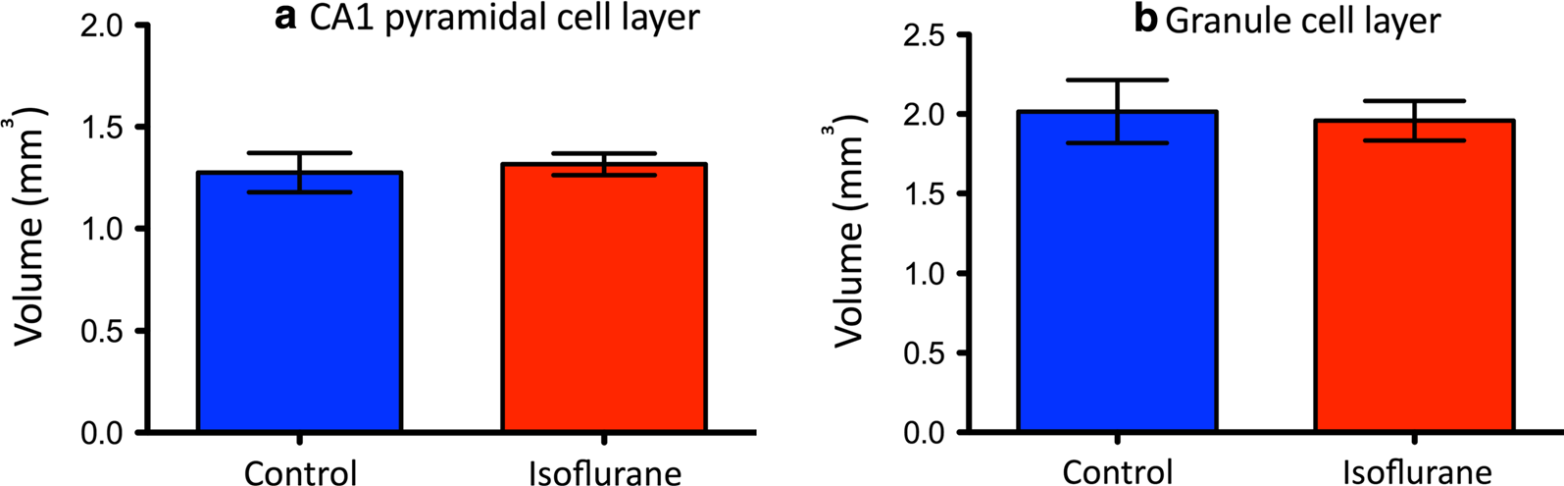
Comparison of the total volumes of the CA1 pyramidal cell layer and the granule cell layer of the dentate gyrus between control and gestationally isoflurane-exposed groups. No significant differences were detected in either of the volumes between the two groups (P = 0.20 and 0.40, **a**, **b**, respectively). Data were analyzed using Student’s t-test and expressed as mean ± S.D

There were no differences in the total volumes of the CA1 pyramidal layer and the dentate gyrus granule cell layer (in mm^3^) between the control and the isoflurane-exposed groups (1.275 ± 0.10 vs. 1.316 ± 0.05 and 2.016 ± 0.20 vs. 1.958 ± 0.12; P = 0.20 and 0.40, respectively) (Fig. 3a, b).

### Discussion

Our results indicate that even a single exposure to a clinically relevant concentration of isoflurane during early gestation causes region-specific hippocampal cell loss that lasts into adulthood. More specifically, in utero isoflurane exposure, at a period equivalent to the human second trimester, causes a persistent decrease in the granule cell number of the dentate gyri of adult male rats. This may, in part, explain the impairment of spatial memory acquisition seen in these rats [12].

The results are, perhaps, not surprising considering that both GABA and glutamatergic transmission, the principal mechanisms of anesthetic action, is primarily involved in early neurodevelopment. It is, therefore, conceivable that an acute modulation of such transmission as would occur with in utero anesthetic exposure could leave a lasting neurodevelopmental imprint. Though our study did not investigate the mechanisms responsible for the reduction in the granule cell number of the dentate gyrus, evidence from other studies suggest a myriad of possibilities. For example, isoflurane is known to cause a dose-dependent decrease in the renewal potential of neural stem/progenitor cells (NPCs) in vitro [16], as well as decreased progenitor proliferation in vivo [15, 17]. Furthermore, isoflurane, when administered alone or in combination during the critical brain growth spurt, is known to cause extensive apoptotic neurodegeneration [1]. At present, the literature on anesthetic-mediated cell death during in utero brain development is conflicting. A study in fetal guinea pigs clearly demonstrated caspase-3 activation and neurodegeneration after 4 h of 0.55% isoflurane anesthesia [18], whereas a similar study done in fetal rats on embryonic day 21 (E 21) with 6 h of 1.3% isoflurane showed a decrease in apoptosis in the hippocampal CA1 region at 2 h following anesthesia [19]. These differences can be partly reconciled by species differences in mammalian neural development. For example, the fetal guinea pig brain is almost fully developed before birth, unlike rats, and hence, anesthetic exposure occurred at the time of synaptogenesis when the developing brain is most vulnerable. This incongruence, nevertheless, introduces an element of caution in interpreting anesthetic neurotoxicity studies from disparate animal models. It is also unclear why isoflurane exposure during 2nd trimester equivalent (E 14) causes a differential, region-specific cell loss in the adult hippocampus, a finding worthy of further exploration. An important limitation of our study is that we did not analyze the brains of female offspring. Though ideal, we limited our analysis to the hippocampi from behaviorally characterized male offspring to correlate morphological changes with behavior. The timeline of the previous study predates the NIH policy on sex as a biological variable, and unfortunately, we did not process the brains of the female offspring at that time.

In summary, our results indicate that gestational exposure to isoflurane causes granule cell loss in the dentate gyrus of adult male rats suggesting that this may, in part, be responsible for the deficits in spatial working memory seen in the male offspring of exposed dams. In a broader sense, our study suggests that anesthetic agents have the potential to disrupt orderly development of the brain and warrants inclusion in the list of various environmental and pharmacological agents that are known to affect normal brain development. This is reinforced by the latest Drug Safety Communication from the Food and Drug Administration (FDA) warning that repeated or lengthy use of general anesthetic or sedative drugs during the third trimester or the first 3 years of life may have consequences for early brain development (https://www.fda.gov/Drugs/DrugSafety/ucm532356.htm). Further studies, therefore, are required to determine the causative mechanisms and to identify anesthetic agents that are safer for obstetric and perinatal use.

## Abbreviations

GABA: gamma amino-butyric acid
CA1: cornu ammonis area 1
E 14: embryonic day 14
NPC: neural stem/progenitor cell
FDA: Food and Drug Administration
